# It Is Imperative to Establish a Pellucid Definition of Chimeric RNA and to Clear Up a Lot of Confusion in the Relevant Research

**DOI:** 10.3390/ijms18040714

**Published:** 2017-03-28

**Authors:** Chengfu Yuan, Yaping Han, Lucas Zellmer, Wenxiu Yang, Zhizhong Guan, Wenfeng Yu, Hai Huang, D. Joshua Liao

**Affiliations:** 1 Department of Biochemistry, China Three Gorges University, Yichang 443002, China; 2 Hormel Institute, University of Minnesota, Austin, MN 55912, USA; yhen@hi.umn.edu (Y.H.); luc.zellmer@gmail.com (L.Z.); 3 Department of Pathology, Guizhou Medical University Hospital, Guiyang 550004, China; ypq1964@163.com; 4 Key Lab of Endemic and Ethnic Diseases of the Ministry of Education of China in Guizhou Medical University, Guiyang 550004, China; zhizhongguan@gmc.edu.cn (Z.G.); wenfengyu2013@126.com (W.Y.); 5 School of Clinical Laboratory Science, Guizhou Medical University, Guiyang 550004, China

**Keywords:** chimeric RNA, *trans*-splicing, reverse transcription, polymerase chain reactions, RNA deep sequencing, expression sequence tag

## Abstract

There have been tens of thousands of RNAs deposited in different databases that contain sequences of two genes and are coined chimeric RNAs, or chimeras. However, “chimeric RNA” has never been lucidly defined, partly because “gene” itself is still ill-defined and because the means of production for many RNAs is unclear. Since the number of putative chimeras is soaring, it is imperative to establish a pellucid definition for it, in order to differentiate chimeras from regular RNAs. Otherwise, not only will chimeric RNA studies be misled but also characterization of fusion genes and unannotated genes will be hindered. We propose that only those RNAs that are formed by joining two RNA transcripts together without a fusion gene as a genomic basis should be regarded as authentic chimeras, whereas those RNAs transcribed as, and *cis*-spliced from, single transcripts should not be deemed as chimeras. Many RNAs containing sequences of two neighboring genes may be transcribed via a readthrough mechanism, and thus are actually RNAs of unannotated genes or RNA variants of known genes, but not chimeras. In today’s chimeric RNA research, there are still several key flaws, technical constraints and understudied tasks, which are also described in this perspective essay.

## 1. Introduction

Classically, mature RNAs in eukaryotic cells are categorized as ribosomal RNAs (rRNAs), messenger RNAs (mRNAs) and transfer RNAs (tRNAs), which are synthesized by RNA polymerases I, II and III, respectively [1]. One of us has recently proposed that noncoding RNAs, which can be processed from RNAs transcribed by either one of the three RNA polymerases, should be considered as the fourth category [1]. However, RNAs can also be classified by other criteria. For instance, while each RNA in any of the abovementioned four categories is derived from a single gene on a chromosome, there are so-called “chimeric RNAs” (herein “chimeras”), which defines those RNAs containing sequences of two genes. Actually, it remains possible that there are trimeric or even tetrameric RNAs, each of which contains sequences from three or four genes, respectively, although until now their existence is only suggested by many expression sequence tags (ESTs), but not by conclusive experimental evidence [2]. As described by some of us before [3,4,5], there hitherto have been tens of thousands of chimeric RNAs of human origin deposited in different databases, with a few examples in the references cited herein [2,6,7,8,9,10], while the entire human genome contains only slightly over 20,000 genes [1,11,12,13,14]. Therefore, mature RNAs can also be dichotomized based on whether they are derived from a single gene or from more than one gene. However, information from chimeric RNA research greatly baffles us in several aspects, largely because there still is a lack of a pellucid definition of chimeric RNAs, which in turn is partly because there is no clear answer to the very basic question “what is a gene?” [15,16,17]. In this essay, we describe our rumination over chimeric RNA, propose a new criterion for defining it to lucidly distinguish chimeras from other RNAs, and expound why we consider that most of the chimeric RNAs reported are inauthentic or should not be esteemed as chimeras. We also describe some common problems, flaws, technical constraints, and understudied tasks in today’s chimeric RNA research.

## 2. Currently, Chimeric RNAs Are Thought to Be Derived from Three Mechanisms

A mature RNA that contains sequences from two genes can be derived from three different mechanisms known hitherto [2,3,4]. The first and well-studied mechanism involves various chromosomal alterations, including DNA insertion, amplification [18], deletion and different types of rearrangement such as translocation. For instance, chromosomal translocation can cause fusion of chromosomes or chromosomal parts, resulting in one or two fusion sites and thus one or two fusion genes, as each fusion site may form a fusion gene. The first and also the best example of the consequence of translocation is the formation of the so-called Philadelphia chromosome observed in 1959 [19,20,21,22,23], which involves chromosomes 9 and 22, i.e., t(9;22)(q34;q11), and results in different *BCL-ABL* fusion genes [24,25]. There have been about 1,000 fusion genes identified hitherto [26], although ten times more are thought to exist, mainly in cancer [27]. In very rare situations, fusion genes can occur in normal human individuals as well, as exemplified by the *TFG-GPR128* [28], *POTE-actin* [29,30,31], and *PIPSL* [32,33] genes. However, this type of fusion gene may actually be regarded as evolutionarily new genes, but not fusion ones [3]. Transcription of fusion genes can be initiated from alternative sites and/or terminated at alternative sites to yield different RNA transcripts, and each of these transcripts can undergo alternative *cis*-splicing to produce different mature RNAs as well. This means that fusion genes, once formed, are expressed and regulated just like other genes in eukaryotic cells with little difference, and therefore it makes more sense to regard their RNAs as regular ones, but not as chimeras.

**Figure 1:**
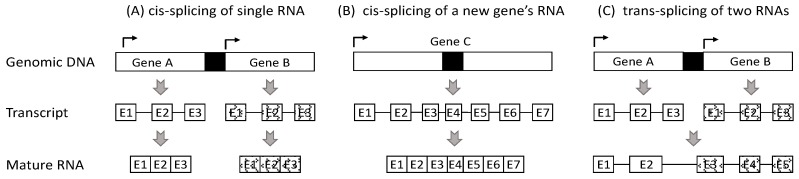
Depiction of how we define different mature RNAs derived from two neighboring genes within the same genomic locus. (**A**) Two genes (genes A and B) are transcribed individually to their own RNA transcripts, each being spliced to a mature RNA. This is a *cis*-splicing procedure; (**B**) Transcription of the upstream gene (gene A) sometimes may not stop at the annotated site but, instead, may go not only into the possible intergenic region (**black box**) but also into the downstream gene (gene B) to produce a much longer RNA transcript that is then *cis*-spliced to a mature RNA, which is considered by us as an RNA of a new, previously unannotated gene (gene C) harbored at this genomic locus; (**C**) Genes A and B are transcribed to their own transcripts that are *trans*-spliced to a single mature RNA, which in our opinion is an unadulterated chimera. (A box with an E and a number inside stands for an exon).

The second mechanism is *trans*-splicing. While *cis*-splicing is a biochemical reaction with one RNA molecule as the substrate and one new RNA molecule as the product (scenarios A and B in Figure 1), *trans*-splicing is a biochemical reaction with two RNA molecules as the substrates but only one RNA molecule as the product (scenario C in Figure 1), which is a chimeric RNA [34]. Obviously, chimeric RNAs derived from *trans*-splicing have no genomic DNA basis [3,4]. *Trans*-splicing occurs often in unicellular organisms [35]. Some mitochondria and chloroplasts of low eukaryotes and plants also use *trans*-splicing to remove discontinuous group II introns [36,37]. Chimeric RNAs have been reported to occur in human cells and in cells of other animals in a physiological situation [5,38,39,40,41,42,43], with examples such as the *KLK4* [44], *Acy1-CoA* and *ACAT1* [45,46] RNAs in humans, the *Dmr* [47] and *Msh4* mRNA variants [48] in mice, as well as some *mdg4* mRNA variants in drosophila [49,50]. The *IGH-BCL2* RNA chimera has been observed in normal spleen [51], whereas *IGH-myc* chimeric RNA has been found in mouse B lymphocytes [52] and in the Peyer’s patch follicles [53]. During the human developmental stage, normal uterine endometrium shows a chimeric RNA formed between the transcript of the *JAZF1* gene on chromosome 7p15 and the transcript of the *JJAZ1* gene at 17q11 [38,40]. Many of these chimeric RNAs in normal cells are thought to be derived from *trans*-splicing events [7,46,50,54,55], which, however, has hardly received unimpeachable experimental evidence, due largely to technical constraints, as described later. In our opinion, *trans*-splicing occurs only as rare events in cells of evolutionarily high animals, and in the physiological situation in humans, the events are probably as scarce as hen’s teeth. However, in carcinogenesis, which is an atavistic process [56,57,58], it may occur more often.

The third mechanism is for those chimeras formed by two neighboring genes on the same chromosome, which is the largest category of chimeric RNAs reported so far but, in our opinion, is also the most debatable one. The ENCODE (the Encyclopedia Of DNA Elements) project once estimated that transcripts from 65% (about two-thirds) of the human genes might be involved in forming chimeric RNAs, but the vast majority of these chimeras involve two neighboring genes on the same chromosome [34,59]. The ENCODE project did not provide mechanistic detail about how the two-neighboring-genes-containing chimeras were formed. However, some studies suggest that at least some of them may be derived from a mechanism of transcriptional readthough [60], i.e., transcription from the upstream gene to the downstream one [3,59], which is illustrated herein as scenario B in Figure 1, such as the *SLC45A3-ELK4* RNA that is present in normal prostate and in prostate cancer [61]. In the database of the National Center for Biotechnology Information (NCBI) of the United States, such RNAs are indicated by using a hyphen to connect the two neighboring genes, as exemplified by the eight noncoding RNAs that are alternatively-splicing products of the *TSNAX-DISC1* transcript (Figure 2 in [62]).

## 3. Most RNAs from Two Neighboring Genes Should Not Be Deemed as Chimeras

An RNA transcribed via the aforementioned readthrough mechanism can actually be regarded in three different ways: (1) it can be considered as an RNA variant of the upstream gene, dubbed herein as gene A, transcription of which does not stop at the annotated site but, instead, reads into and stops at the downstream gene, designated herein as gene B; (2) it can be considered as an RNA variant of gene B, transcription of which is initiated from an alternative site upstream of the annotated one; and (3) it can be deemed as an RNA transcript of an unannotated gene harbored at this genomic locus, herein referred to as gene C. We favor the third scenario, since it has been a well-accepted notion that “one gene may contain one or more other genes” with many examples in the NCBI database (some example illustrations are copied from the NCBI and shown in [1,62]). However, we have no objection to the first two scenarios that are actually the situations of alternative initiation and alternative termination, respectively, of transcription, mechanisms of which have all been well articulated in the literature for many genes. In any of these three scenarios, RNA transcripts may undergo regular *cis*-splicing, showing no any obvious difference from transcripts from already-annotated genes [63,64]. We favor the third scenario because these unannotated, i.e., newly discovered genes have no difference from, and should be studied like, already-annotated genes at all levels, including the levels of transcription, post-transcription, translation, post-translation, and protein transportation, although some of these genes may be noncoding and are not regulated at some of these levels, such as the *TSNAX-DISC1* gene (Figure 2 in [62]). We strongly argue against the currently dominant concept in the chimeric RNA research that esteems the *cis*-splicing-derived mature RNAs from these unannotated genes as chimeras. This is because considering these RNAs as chimeras implies that these genes are different from the vast majority of already-annotated ones, thus misleading and encumbering their characterization at some of these levels, although it sounds more novel and more easily brings us grant supports and publications. On the other hand, considering most of these with two neighboring genes as individual unannotated ones also implies that are many more human genes than just approximately 20,000 genes as we currently thought [11,12,13,14], thus making more sense.

Theoretically, two neighboring genes may be transcribed separately to produce their own RNA transcripts in the regular way, but the two transcripts are then *trans*-spliced to an RNA, which is an unadulterated chimera (scenario C in Figure 1). The problem is that, although there have been many mature RNAs known to contain sequences of two adjacent genes, few, if any, studies provide cogent mechanistic detail about whether these RNAs are formed via *cis*-splicing of a single RNA transcribed via a readthrough mechanism or are formed via a *trans*-splicing of two RNA transcripts. In some studies that claim the occurrence of a *trans*-splicing event, little mechanistic detail and experimental evidence about such event per se have actually been given. Therefore, there is no way of knowing which of these reported two-neighboring genes-containing RNAs are authentic chimeras and which others are just *cis*-splicing-derived regular RNAs of unannotated genes, mainly because there is no feasible technique to determine RNA transcripts before splicing. Splicing occurs immediately after transcription is initiated [65], and is finished almost at the same time of transcription termination [66], leaving us only a small window of time to sift out the product RNAs from the substrate RNAs during a splicing procedure, especially for many genes in a high throughput manner.

## 4. There Are Different Ways to Catalog Chimeric RNAs

**Figure 2:**
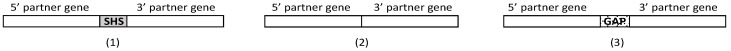
Three different types of chimeric RNAs classified based on the relationship between the two partner sequences. SHS, short homologous sequence; GAP, an unmatchable sequence.

Chimeric RNAs can be sorted to different subgroups by different criteria, as recounted before [2,3,4]. Besides the criteria delineated above, a further criterion is the relationship between the two gene elements, herein referred to as two partners, of a chimera [2,8]. Based on this relationship, chimeras can be sorted to three groups, as illustrated in Figure 2. One group contains those in which the two partner sequences are reversely complementary to each other at a region called “short homologous sequence (SHS)”. Another group includes those in which the 5′ and 3′ partner sequences are directly connected. The third group includes those in which there is a sequence inserted between the two partners that cannot be matched to any genomic region and is thus called a “gap”. Actually, for the putative trimeric or tetrameric RNAs, the relationship between any two neighboring genes’ sequences should also be one of the three situations [2].

## 5. RT or PCR Creates Many Artifacts that Fabricate “*Trans*-Splicing”

Most known DNA polymerases require a DNA or RNA oligo as a primer to initiate the DNA synthesis, although some others use a protein to prime [67,68]. DNA polymerases are widely used in nucleic acid research. For instance, reverse transcriptases (RTases), such as those derived from the Avian myeloblastosis virus (AMV) [69] and the Moloney murine leukemia virus (MMLV) [70,71], as one type of DNA polymerase use tRNA as the primer in the parental retroviruses but can also use DNA primers in reverse transcription (RT) in vitro. On the other hand, Taq DNA polymerase is often used in polymerase chain reactions (PCR) with DNA primers, which often follows RT in molecular cloning procedure.

**Figure 3:**
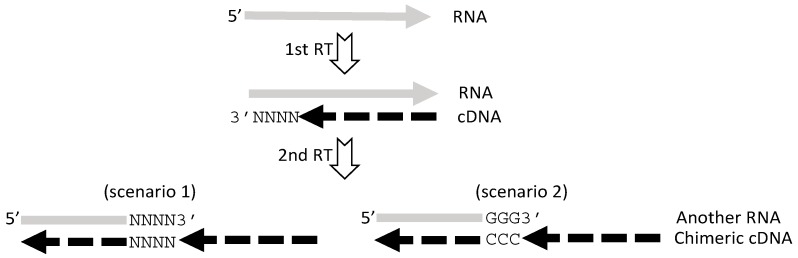
Depiction of artificial chimeric cDNA created by hypothetical “consecutive RTs”. In RT (1st RT), an RNA is converted to the first strand of cDNA with its last several Nts coined as “NNNN”. An RNA of another gene may have its first several Nts reversely complementary to these NNNN and thus can anneal to the cDNA end, which allows RT to continue (2nd RT), resulting in an artificial chimeric cDNA (scenario 1). Most RTases append additional Nt or Nts in a non-template manner. For instance, MMLV RTase usually appends CCC or GGG, allowing any RNA with the first Nts being GGG or CCC to anneal to the cDNA end to create a chimeric RNA in the 2nd RT (scenario 2). For detail, see Reference [3]. Arrows point to the 5′-to-3′ direction.

Lerat et al. [72] and Tuiskunen et al. [73] have once surmised that artifacts may occur in the following situations during an RT-PCR procedure: (1) RTase may be carried over from the RT system to the PCR tube wherein it allows RT to continue with the PCR primers as the primers and with the first strand of cDNA as the template; (2) when RNAs are carried over from the RT system to the PCR tube, Taq may have an RT activity and convert RNA to cDNA; and (3) the 3′ end of the first cDNA strand can loop back to form a hairpin structure and prime the synthesis of the second cDNA strand during the PCR. The first scenario would actually be consecutive RT reactions, as one of us phrased previously [3], for one of two basic reasons: First, if the first cDNA strand has its last several nucleotides (Nts) reversely complementary to the end of another RNA or cDNA, these Nts can anneal to this RNA or cDNA and use it as the template for the RT to proceed (scenario 1 in Figure 3). Since all DNA or RNA sequences are formed by only four bases, a several-Nt homolog (in a reversely complementary manner) should be common in the 3′ ends of numerous DNA or RNA shreds in a reaction tube. This is why usually in vitro RT does not really require the presence of random hexamers or even a poly-dT primer, because the RNA sample contains numerous RNA or DNA shards as endogenous random primers [3,69,70,74,75,76,77,78,79,80]. Second, most, if not all, DNA polymerases can append one or several Nts at the end of the newly synthesized DNA in a non-template manner [81,82,83,84,85,86], as one of us summarized before [3]. MMLV RTase as the most commonly used enzyme in RT more often appends GGG or CCC at the cDNA end [87,88,89,90,91,92], which can anneal to any RNA or DNA sequence ending with CCC or GGG to allow the RT to elongate (scenario 2 in Figure 3). If it is an RNA that anneals to the cDNA, an RNA–cDNA chimera is created as the first strand but then the second strand is a DNA chimera, synthesized in the first cycle of the ensuing PCR either by Taq or by the carried-over RTase [3].

Artifacts can also occur in PCR with production of many single-stranded DNA oligos or double-stranded DNA fragments that are shorter than the anticipated amplicon, due to aborted DNA polymerization. The last several Nts of these shorter DNA oligos or fragments may anneal to another gene’s cDNA and serve as the template for the DNA synthesis to continue, yielding a chimeric DNA fragment [93]. This first-strand of chimeric DNA may be elongated to a much longer sequence in the subsequent cycles of PCR.

**Figure 4:**
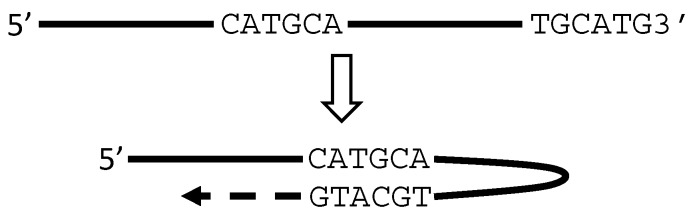
Possible formation of spurious “*trans*-splicing” of sense and antisense transcripts. If an RNA or DNA end has several Nts (say, TGCATG) reversely complementary to a 5′ region (say, CATGCA), it may loop back to anneal to this region and allow RT to continue with the 5′ region as the template. As a result, the cDNA contains not only the sense sequence but also the antisense (in dash arrow) sequence, thus becoming a spurious chimera. Examples can be found in Reference [4].

16S rRNA, which is commonly used in molecular surveys of bacterial and archaeal diversity [94,95,96,97,98], has been well known to engender many chimeric cDNAs during cDNA library construction and the ensuing deep sequencing [93,99,100,101,102,103,104,105,106,107,108], which serve as good examples of fakes. A few 16S-rRNA-containing chimeras have also been reported in human and mouse cells [109,110,111,112,113,114,115,116,117], which are repeatedly detected and well-studied but in our opinion are technical artifacts [4]. They are frequently detected partly because in human and mouse cells the 16S rRNA is encoded by the mitochondrial genome that not only has hundreds or even thousands of copies in a single cell but also contains many reversely complementary regions [4]. These regions allow an RNA to loop back to form a hairpin structure, which allows the RT to elongate with the 5′ sequence as the template, creating a cDNA that looks like a product of *trans*-splicing of the sense and antisense transcripts (Figure 4). This sequence property of many 16S-rRNA-containing chimeras suggests to us that, if in a chimera one of the two partners has its sequence at the junction reversely complementary to a 5′ region of its parental RNA, it should alert us about the possibility of the self-priming derived spuriousness, as elucidated in more detail previously [4]. Transcription of 16S rRNA has been reported to be terminated at many sites [118,119], and these many ends of the RNAs create many opportunities for the scenarios illustrated in Figure 3 and Figure 4 to occur [4].

The above described self-priming by looping back often occurs in retroviruses [120], retroplasmids [121] and some bacteria [122]. The stem part of the hairpin structure formed via looping-back, which serves as the primer, should be short, because the reversely complementary region at the 3′ end of an RNA or DNA is usually short. A question is thus raised as to how short an RNA or DNA oligo can be a functional primer to initiate DNA synthesis. Unfortunately, this question has received much less attention so far and has not yet had a clear answer, but it may vary among different situations and depend on the DNA polymerase. RTases of the AMV and MMLV origins have been reported to use 15–18 Nts of a tRNA as a primer, and RTases from other retroviruses, such as the human immunodeficiency virus (HIV), also use similar numbers of Nts in tRNA as primers [123,124,125,126,127]. However, it is possible that the HIV RTase may only need a primer of 9 Nts [128], and it has also been reported that AMV RTase only requires a 3-Nt or 4-Nt DNA primer to prime DNA synthesis [129]. It has been reported that Taq requires a primer of, or longer than, 9-Nts, and for this reason some peers consider that Taq should not be able to act as an RTase in PCR even if some random hexamers are carried over from the RT system to the PCR tube [130]. One of us recently showed that a 4-Nt homolog might be able to prime DNA synthesis with an MMLV RTase and a Taq in a common RT-PCR procedure [4]. It is clear that random hexamers are sufficient to prime RT in vitro, as it is a routine lab practice. Of course, the efficiency of a primer depends not only on the length of the oligo but also on which bases that are in the primer and on the temperature provided for the annealing, which usually varies among different PCR procedures. Moreover, it is worth mentioning that unlike DNA polymerases, some RNA polymerases can initiate de novo RNA synthesis in a primer-independent manner, besides the back-priming like DNA polymerases [131], but whether this property may also create artifacts remains unknown.

## 6. There Are Other Artifacts with Unknown Mechanisms

While those chimeric RNAs in which one partner sequence is directly linked to the other without an SHS or a gap, i.e., those type 2 chimeras in Figure 2, may be artifacts created by a ligase during construction of cDNA libraries, the reason for the appearance of many cDNAs that contain a gap, as seen in many ESTs [2], is completely unknown. *Trans*-splicing should not be able to create such gap sequences that are completely unmatchable to the genomic DNA of any known organism deposited in the NCBI database [2]. Actually, there are many cDNA sequences, especially seen in RNA deep sequencing results, which cannot be matched to any organism’s genome in the NCBI database either. It is possible that these unmatchable sequences occur as artifacts in RT or PCR and may contribute to formation of artificial chimeric RNAs, although this hypothetic thinking still lacks convincing evidence. The gap sequences, which can be over one kilo-base pairs in many chimeric ESTs [2], may be good examples for the possible existence of unknown mechanisms for creating artifacts.

## 7. Currently, cDNA Protection Assay Is the Best Approach for Verification of Chimeric RNAs

**Figure 5:**
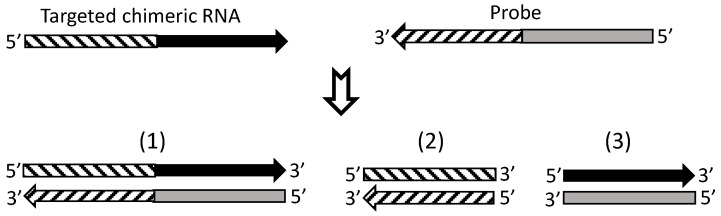
Illustration of how RNA protection assay, in situ hybridization or Northern blotting may produce a false signal by detecting only one of the two partner sequences. One chimeric RNA contains two-partner-genes’ sequences indicated by a striped bar and a black bar, respectively. In an RNA protection assay, in situ hybridization or Northern blotting, an RNA probe that contains sequences reversely complementary to the two partner-genes’ sequences is used to hybridize with the target chimeric RNA (scenario 1), followed by use of an enzyme to digest away single-stranded excessive probe and irrelevant RNAs. However, in the cells one or both of the two partner genes may also be expressed to RNAs that can also hybridize to part of the probe (scenarios 2 or 3) to form an unwanted hybrid, which is a noise. Since these techniques do not provide sequence information of the hybrid to confirm its identity, there is no way of knowing whether the resultant hybrids belong to the scenario 1, 2 or 3 or to some combination of the three.

Most of the tens of thousands of chimeric RNAs deposited in different databases have not yet been verified with any bench technique. Nevertheless, a small portion of reported chimeras have been verified using RT-PCR with or without confirmation by sequencing the cDNA, while an even smaller number of them have been authenticated using RNA protection assay, Northern blotting and/or in-situ hybridization. However, although these latter techniques have their merits and are more reliable than RT-PCR, none of them are authoritative enough to corroborate the true existence of a chimeric RNA. RNA protection assay does not involve RT or PCR amplification and thus will not create those RT- or PCR-related artifacts narrated above. However, because it does not involve amplification, its sensitivity is low and it can only detect those chimeras that are highly expressed. Moreover, the sequence of the probe used needs to be carefully designed or otherwise it can lead to data misinterpretation. The biggest weakness of RNA protection assay is that it does not provide sequence information of the protected RNA and thus cannot confirm that both partner sequences of a chimera have been protected. Actually, it is highly possible that only one of the two partner sequences is protected (Figure 5). Northern blotting has the same weaknesses as RNA protection assay and is less sensitive. In-situ hybridization assay has the same weakness as well, especially because it lacks sequence data to prove that the hybrid contains both, but not just one of the two, partner sequences (Figure 5).

Attempting to correct the weaknesses of the abovementioned methods, we have modified the RNA protection assay to a cDNA protection assay in which it is the cDNA, but not the parental RNA, that is protected [79]. In this assay, an aliquot of RNA is reversely transcribed to the first strand of cDNA. If an MMLV RTase, but not its mutant (MMLV-M), is used, inactivation of its RNase H activity ensues. The cDNA is then used as the probe to hybridize with another aliquot of the original RNA to form a RNA–cDNA hybrid. With its RNAase H activity inactivated, the carried-over RTase should not be able to digest the RNAs. After an S1 endonuclease is used to digest away irrelevant RNAs and all the excessive single-stranded cDNA probe, the RNA–cDNA hybrid is amplified using PCR with Taq, followed by DNA sequencing to confirm the identity of the protected cDNA. Compared with its parental RNA protection assay, this cDNA protection assay has two advantages, i.e., offering PCR amplification and DNA sequencing. Although this method involves RT that will likely create some bogus cDNA chimeras, the fakes will not be protected by unadulterated chimeric RNAs and will thus be chopped up by the S1.

## 8. We Propose New Criteria to Classify Chimeric RNAs

We propose to use “fusion RNA” to describe those RNAs transcribed from fusion genes, and to use “chimeric RNA” to describe those RNAs that are authentic chimeras derived from amalgamation of two RNA transcripts, via *trans*-splicing of two RNA molecules or via other currently unknown mechanisms, such as the hypothetical “transcriptional jump” and “chromosomal interaction” [132]. For those RNAs containing sequences of two adjacent genes on the same chromosome but sans a knowledge of how they are formed, we can temporarily put them under the umbrella of “readthrough RNA”. Of these RNAs, those that are later confirmed to be transcribed from unannotated genes via a readthrough mechanism should be excluded from this “readthrough RNA” category and deemed as RNAs of newly discovered regular genes that await annotation (scenario 2 in Figure 1). Since readthrough genes in the NCBI are indicated with a hyphen to join two neighboring genes together, as exemplified by the *TSNAX-DISC1* (Figure 2 in [62]), the simplest way to annotate them is to follow this way of the NCBI. Those that are later confirmed to be products of *trans*-splicing of two individual transcripts (scenario 3 in Figure 1), or are formed via a currently unknown mechanism that joins two RNAs together, should also be removed from the “readthrough RNA” category but put into the authentic chimera family. In a nutshell, only those RNAs that lack a fusion gene as a genomic basis and are derived from two individual RNA molecules should be considered veritable chimeras, whereas fusion genes and unannotated genes are actually expressed and regulated in the same way as classically annotated genes, and thus there is no reason to consider them special.

## 9. There Are Other Flaws, Constraints and Understudied Tasks in Chimeric RNA Research

Most reported chimeras are “identified” using high-throughput sequencing or a bioinformatic analysis of ESTs, or coalescence of the two, and are dubbed by us as “putative chimeras” because they have not yet been verified. Besides those already described in the above sections, in our meditation today’s chimeric RNA research still has several other major flaws, technical constraints and understudied tasks:

1. There are still thousands of putative chimeric RNAs that contain sequences of two genes on two different chromosomes [27]. For most of them, it is still unclear whether they have a fusion gene as the genomic basis, and thus it is still unclear whether they are genuine chimeric RNAs by our definition.
2. Many RNAs are claimed to be derived from a readthrough mechanism but actually lack concrete experimental evidence proving the readthrough, since detection of a long pre-RNA transcript is one thing but causally-linking it to the mature RNA that contains two-gene sequences is another thing. Therefore, the possibility for them to be derived from a *trans*-splicing mechanism and thus to be genuine chimeric RNAs still exists, which needs to be ruled in or out.
3. Some chimeric RNAs have been reported to be recurrent, usually in cancer. However, often it is not clear whether the “recurrence” means that it is exactly the same chimeric sequence that appears repeatedly, or it means that the same two genes produce chimeric RNAs that are highly similar but still differ slightly in sequence. This question is raised because we sometimes have inquired of peers about the “recurrent” chimeric RNAs they observed and knew that the repeatedly detected chimeras differed slightly among each other in sequence. The reason for the small difference in sequence should be determined.
4. Technical detail is insufficiently discussed in many published studies of chimeric RNAs, especially about how possible it is that the chimeras are spurious. In our opinion, when describing a new chimeric RNA, more-than-usual technical detail needs to be furnished and, moreover, it needs to address: (1) whether it has a fusion gene as a genomic basis; and (2) whether it contains an SHS or a gap sequence that highly indicates an artifact.
5. Some chimeras encode proteins that share part of the sequence with the proteins produced from one or both of the parental genes. Determining whether these chimeras have protein products is technically difficult in most cases, due to the lack of fusion-protein specific primary antibodies, which in turn is due to a technical constraint in raising such antibodies. Antibodies raised via a traditional approach will likely recognize proteins from one or both parental genes. Theoretically, antibodies for the proteins from one of the two parental genes may be available and be used in western blotting to distinguish fusion-proteins from the proteins of the parental genes by their difference in molecular weight. However, since most genes produce multiple protein isoforms [62], as one of us has shown for protein products of many genes [133,134,135,136,137,138], in practice it is difficult to use these antibodies to corroborate the true existence of protein product(s) of a chimeric RNA, especially with an immunohistochemical staining approach that does not allow us to distinguish one protein from the others by their molecular weights.

## 10. Concluding Remarks

The overarching theme of this perspective essay is that chimeric RNA is still ill-defined and many of those chimeras deposited in various databases are likely to be technical artifacts or should not be regarded as chimeras, which may sound provocative to some peers. We propose that only those mature RNAs formed by joining two RNA molecules together via *trans*-splicing or some currently-unknown mechanisms without a fusion gene as a genomic basis should be authentic. Many of those containing two adjacent genes’ sequences are probably mature RNAs of newly discovered genes awaiting annotation, or are RNA variants of one of the two parental genes, but are not chimeric RNAs. Since there have already been tens of thousands of putative chimeric RNAs reported and the number is still soaring in the literature, it is imperative to clearly define chimeric RNAs and vigorously verify them. Otherwise, such a larger number of putative ones will not only mislead the chimeric RNA fraternity but will also hamper cloning and characterization of those fusion genes and those newly discovered genes. Actually, most fusion genes have not yet been fully characterized to such a detail as for the *BCL-ABL* fusion genes on the Philadelphia chromosome, while most two-neighboring-genes-containing RNAs have not yet been fully studied at the transcription and other steps. On the other hand, at present, *trans*-splicing is the only known mechanism for formation of authentic chimeras, but it may not be commonly used in human cells, meaning that either genuine chimeras in human cells are many fewer than many peers think or other unknown but commonly used mechanisms exist and await unearthing.
